# Lipid transport mechanisms in human ABCA family transporters: a structural perspective

**DOI:** 10.1042/BST20250138

**Published:** 2026-05-07

**Authors:** Subhrajyoti Dolai, Amer Alam

**Affiliations:** The Hormel Institute, University of Minnesota, Austin, Minnesota 55912, U.S.A.

**Keywords:** ABC transport proteins, ABCA1, ABCA7, apolipoproteins, membrane proteins, membrane transporters

## Abstract

ATP-binding cassette (ABC) transporters are essential membrane proteins that couple ATP hydrolysis to move diverse substrates across lipid bilayers through large-scale conformational changes. In humans, 48 ABC transporters span seven subfamilies (A–G); within these, the ABCA subfamily mediates cellular lipid handling in contexts ranging from neural function to pulmonary surfactant production, and its dysfunction contributes to human disease from cardiovascular disorders to Alzheimer's. These diverse physiological roles all depend on precise lipid translocation within or across membrane systems, a shared principle that is often underemphasized in broad “lipid-transporter” classifications. This review summarizes the structural landscape of the ABCA family and re-examines the mechanistic insights that have emerged. We compare and contrast transport models derived from detergent-solubilized and lipid-embedded structures, with particular emphasis on lipid-embedded ABCA7, which supports a membrane-integrated mechanism in which the bilayer itself contributes to the transport pathway. We highlight shared rigid-body transitions, outline open questions surrounding transport directionality and protein–lipid coupling, and suggest that future models should treat the membrane not merely as a passive scaffold but as an integral component of the transport mechanism, while recognizing that membrane-integrated behavior is currently established structurally only for ABCA7 and remains a working hypothesis for other family members.

## Introduction and disease relevance

The ABCA subfamily of ATP-binding cassette transporters plays structurally and physiologically distinct roles in human biology, including cholesterol efflux, pulmonary surfactant production, photoreceptor retinoid clearance, and lipid handling in phagocytic cells [1–7]. The 12 known members (ABCA1–10, 12–13, with ABCA11 being a pseudogene) can be broadly grouped in two distinct categories: those for which clear structure–function data are available and those where functional ambiguities and lack of experimental validation hamper pinpointing their physiological roles. The former includes among the best characterized of the family: ABCA1, which facilitates phospholipid and cholesterol efflux to apolipoproteins like apolipoprotein A1 (apoA1), catalyzing HDL formation through an extracellular domain (ECD)-integrated hydrophobic tunnel [8–11]. It prefers phosphatidylcholine over phosphatidylserine [8] and is expressed in liver, brain, adrenal glands, placenta, and macrophages [12–14]. ABCA1 mutations cause Tangier disease, a rare HDL-deficiency syndrome, and familial HDL deficiency [15–17] and are linked to impaired lipid efflux [18,19]. ABCA2 is expressed in oligodendrocytes, regulates ceramide metabolism and lysosomal cholesterol sequestration [20–24] and is linked to amyloid precursor protein (APP) processing and amyloid-beta (Aβ) generation [25,26]. ABCA3, expressed in alveolar type II lung cells and macrophages, loads surfactant phospholipids—primarily short-chain phosphatidylcholine—into lamellar bodies [27–31] and exports miltefosine and free cholesterol [32,33]. Mutations cause neonatal respiratory distress syndrome and interstitial lung disease [34–39]. While all other ABCA transporters are exporters, ABCA4 occupies a unique place with opposite substrate transport directionality. This import functionality is, with the exception of the lysosomal cobalamin transporter ABCD4, unique among all human ABC transporters. ABCA4 is specific to rod and cone photoreceptors [40,41], and flips N-retinylidene-PE from the luminal to cytoplasmic leaflet [8,42–47]. Disease mutations cause Stargardt disease, the most common form of inherited juvenile-onset macular degeneration, and other retinal degenerations [48–50]. ABCA5 is widely expressed, including in skeletal muscle, liver, kidney, and CNS regions such as the hippocampus and frontal cortex, and is associated with reverse cholesterol transport and amyloid β production [51,52]. ABCA7, enriched in microglia and neurons, promotes phospholipid and cholesterol efflux, presumably through interactions with apoA1/apoE [8,53–72], regulates ceramide homeostasis in keratinocytes [73], and mediates Aβ clearance by microglia [64]. ABCA7 is genetically linked to Alzheimer’s disease risk, with multiple coding and noncoding variants implicated [58,74–76]. ABCA12, expressed in granular layer keratinocytes [77–79], transports glucosylceramides into lamellar granules and modulates protease delivery [80] and is essential for skin barrier formation. Its loss-of-function variants causing severe congenital keratinization disorders with impaired barrier and generalized scaling, including Harlequin ichthyosis, lamellar ichthyosis, and congenital ichthyosiform erythroderma [80–84].

The second group includes the lesser characterized ABCA6, ABCA8, ABCA9, and ABCA10. While expressed in lipid-handling tissues and potentially relevant to metabolic regulation, they lack resolved structures, defined substrates, or clear disease links. Their co-localization on chromosome 17q24 [85–88] and overlapping expression profiles suggest functional redundancy or conditional specialization. While they are expressed in lipid-handling tissues (e.g., liver, muscle, macrophages), they lack definitive biochemical or substrate-transport data. Most findings are correlative or transcriptomic. Unlike ABCA1, ABCA3, ABCA4, ABCA7, and ABCA12, which are directly tied to well-characterized diseases, these four have only tentative links—mostly from genome-wide association studies (GWAS) or expression profiling in metabolic or inflammatory conditions. Their substrate profiles, regulatory roles, and evolutionary pressures appear similar—suggesting potential redundancy or conditional expression. Future studies may reveal whether these transporters represent backup systems, regulatory buffers, or unrecognized lipid-specific exporters. Finally, ABCA13, the largest of all ABC transporters, comprising over 5000 amino acids, while implicated in psychiatric and neurodevelopmental disorders, remains poorly understood mechanistically and specifics of its molecular structure, membrane trafficking, and biochemical function remain largely unexplored [89,90].

## Structural hallmarks and domain topology

**Figure 1:**
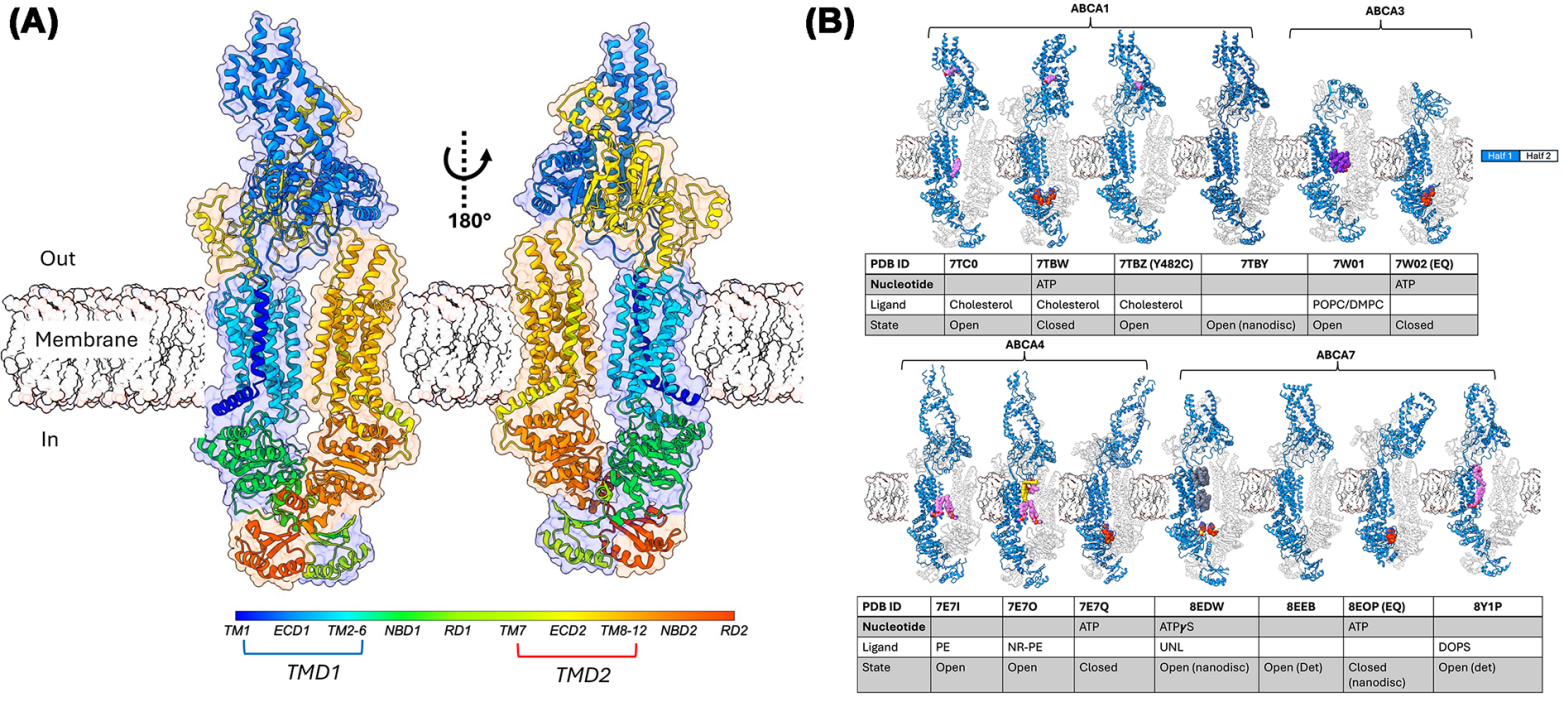
Overall structure, domain arrangement, and conformational spectrum of ABCA family transporters (**A**) cryo-EM structure of human ABCA1 in its detergent-purified apo state with individual domains depicted in rainbow coloration from the N to C termini, with color key shown. TMDs, each containing six TMs (with the exception of ABCA10, which is predicted to have a truncated TMD missing TM1), are shown, with ECD1 and ECD2 intervening between the first two TMs of each. The two halves of the transporter, shown enveloped (transparent surfaces) and colored blue and orange for half 1 and half 2, respectively, highlight ECD- and RD-mediated domain swapping. (**B**) Structures of ABCA1, ABCA3, ABCA4, and ABCA7 shown in ribbon format with modeled substrates/ligands shown in sphere representation.

Advances in cryo-EM over the last decade have heralded tremendous progress in our understanding of the domain arrangement and conformational spectra of ABCA transporters [91–100]. The overall architecture was revealed by the structure of human ABCA1 (Figure 1A), showing two non–domain-swapped transmembrane domains (TMDs) characteristic of the Type II ABC exporter/Type V ABC transporter fold and bearing distinct similarities to those of the ABCG family transporters [101–103]. However, the first two helices of each six-transmembrane bundle (TM1–TM2 and TM7–TM8) are separated by large ECDs that co-fold into a distinct three-tiered structure divided into a base, tunnel, and stalk. C-terminal to each TMD are canonical nucleotide-binding domains (NBDs), each terminating in short regulatory domains (RDs). Each ECD makes extensive contacts with its opposite ECD and TMD. Similarly, RD1 and RD2 are conserved and participate in cross-domain stabilization—with each RD contacting the opposite NBD, mimicking the domain-swapping behavior of the ECDs across TMDs. ABCA1 RDs were initially modeled incorrectly (5XJY) due to poor resolution and later corrected (PDB 7ROQ) [104] based on structures of human ABCA4 [97]. These contacts may underlie transport cooperativity, but their functional role is not yet defined. Overall, the RDs complete the domain-swapped lattice, potentially coupling NBD/RD motions to ECD reorientation. The ECDs form hydrophobic tunnels proposed to facilitate lipid capture or extrusion. Subsequent structural work on other ABCA transporters confirmed this conserved TM1–ECD1–TM2–6–NBD1–RD1–TM7–ECD2–TM8–12–NBD2–RD2 domain arrangement.

While TMDs and NBDs show family-typical sequence and structural conservation, the ECDs are far more divergent. Qualitatively, ABCA1, ABCA4, and ABCA7 share “canonical” large ECD1 modules that form extended tunnels; ABCA3, ABCA5, ABCA6, and ABCA8–10 carry shorter, more compact ECDs; and ABCA2, ABCA12, and especially ABCA13 possess abnormally large ECDs. ECD2 is uniformly shorter in all paralogs and moderately conserved but still tracks these broad categories. AlphaFold predictions, while most reliable where experimental structures are available, generally mirror this pattern and resemble the conformations seen for detergent-purified transporters. Notably, ABCA10 is predicted to contain only five transmembrane helices in TMD1 with its N terminus emerging in ECD1—an anomaly that may reflect non-canonical folding, misprediction, or a true divergence from the family norm. In short, the TMD–NBD engines are conserved while the ECDs encode divergence. This implies that diversification of extracellular gate geometry, rather than catalytic core evolution, may dictate substrate context and interaction partners.

## Conformational spectrum and insights into transport mechanisms

**Figure 2:**
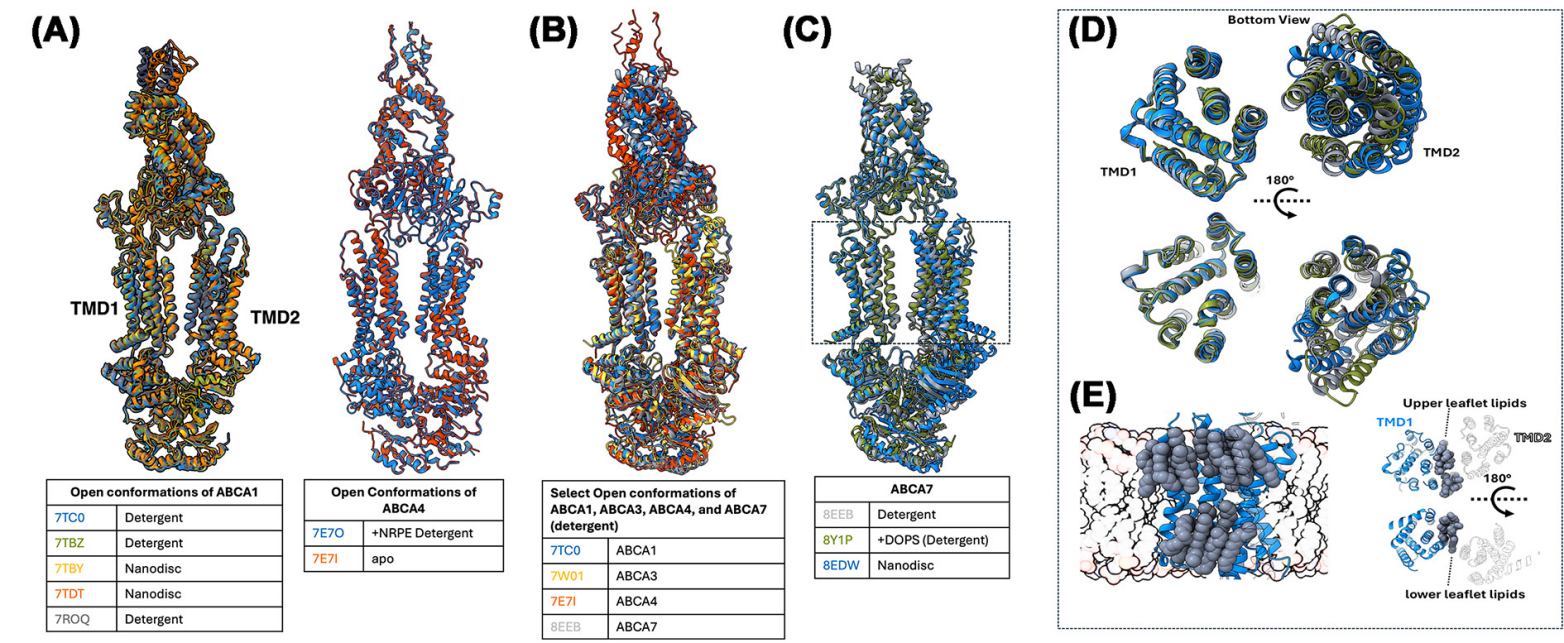
Comparison of open conformation structures of ABCA transporters (**A**) Overlay of ABCA1 (left) and ABCA4 (right) structures with PDB IDs and purification state indicated, emphasizing the shared adopted architecture of the TMDs between different apo and substrate-bound states. Note: Multiple structures for near-identical states of ABCA4 are available but excluded here for visual simplicity. (**B**) Overlay of select detergent-purified ABCA1, ABCA3, ABCA4, and ABCA7 structures in detergent-purified form, emphasizing the shared adopted architecture of the TMDs. (**C**) Overlay of detergent-purified and nanodisc-embedded ABCA7 structures showing subtle deviations of TMD2 between apo and DOPS-bound states in detergent and its nanodisc-embedded open state. (**D**) Bottom (cytoplasmic) and top (exoplasmic) view of the ABCA7 TMDs corresponding to the boxed region from panel (E). (**E**) Zoomed-in views of boxed-in panel (A) showing the nanodisc-embedded open conformation of ABCA7 viewed from the side, with the second half of the transporter clipped for clarity to show upper- and lower-leaflet acyl chains representing the bilayer leaflet patch (left) and upper and lower leaflet lipids viewed from above or below the membrane plane, respectively.

All ABCA transporter structures solved in detergent capture a limited conformational spectrum dominated by rigid-body shifts between nucleotide-free outward-facing states and nucleotide-bound closed states, regardless of the presence of externally added transport ligands. As highlighted in Figures 1B and 2A–C, the structural clade of ABCA1, ABCA3, ABCA4, and ABCA7 all show nearly identical TMD/NBD conformations. The TMD bundle and the α-helical subdomain of each NBD move as a single unit. Only the RecA subdomain undergoes the expected reorientation upon ATP binding. This constitutes a conserved mechanistic engine shared with the ABCG family [98,105,106], distinct from the hinge-type conformational diversity of Type I exporters (ABCB/C/D), exemplified by ABCB1 [102]. The ECDs (ECD1 and ECD2) likewise behave as a coupled rigid body, and the core of each RD is rigid across states, with only the NBD-coupling/peripheral helices exhibiting secondary reorientations between open and closed conformations. These rigid body groups (RBGs) define the mechanical backbone of ABCA motion and underlie the global conformational transitions of the substrate transport cycle. These conformational states superficially align with a modified alternating-access framework, sometimes termed the “lateral access and extrusion” model, in which the ATP-free state presents a V-shaped TMD cavity that is more expanded at the extracellular leaflet, and NBD dimerization couples to TMD closure. Despite structural resolution across different liganded states, the mechanism is often depicted in a formulaic way: substrate binds, NBDs close, substrate is expelled, and NBDs reset. Substrate binding is inferred from comparison of detergent-solubilized transporters with and without extraneously added, generally in large molar excess, lipidic substrate. Mechanistic cartoons often echo lock-and-key simplifications used to explain substrate translocation in both exporters and importers like ABCA4 despite identical conformational spectra, with substrate density for ABCA4 observed in the upper leaflet [95–97], that for ABCA3 in the lower leaflet [94], and that for ABCA7 in both leaflets [98,99]. This rigidity, while chemically plausible, risks masking subtler lipid-coupled behaviors where the membrane is not a passive solvent but the compressible, elastic component of the overall transport machinery coupling nucleotide-coupled domain motion to leaflet distortion.

An alternate substrate translocation mechanism has been proposed for the human ABCA7 transporter, whose structures in lipid-embedded form in the presence of a non-hydrolysable ATP analog depart from this pattern [98]. In its open state, a continuous patch of bilayer enters the TMD core, which adopts a wider conformation than the state seen in detergent, the latter suggested to be an intermediate open state (Figure 2D). MD simulations with restrained protein coordinates show spontaneous passage of PE/PC through this cavity, implying a transient but coherent lipid path. Upon ATP binding and NBD closure, this path collapses. This geometry provides compelling evidence of conformational cycling of the NBDs driving shear and wedge motions that transiently reshape this through-passing bilayer—altering local order, leaflet registry, and curvature—to bias specific phospholipids toward the extracellular interface. The large ECDs may therefore function as capture and presentation platforms, coupling membrane deformation to accept or transfer lipids. The model is compatible with both lipid transfer from the inner to outer leaflet and extrusion of outer leaflet lipids upon TMD closure. The latter property has also been attributed to ABCA1, argued to be an outer/upper leaflet lipid extruder [9].

Interestingly, structures of ABCA1 in nanodiscs revealed an identical conformation to that observed in detergent, pointing to a distinct conformational spectrum than that of lipid-embedded ABCA7 [92,93]. It is important to note, however, that in contrast to those of ABCA7, these structures were determined in the absence of nucleotides. A second, independent ABCA7 study reported apo and phosphatidylserine (PS)-bound structures in detergent/mixed micelles, as well as PS-enriched apoE-containing particles in functional assays [99]. The apo detergent structures closely resemble the original digitonin ABCA7 structures and the detergent-based conformations of ABCA1/3/4, while the PS-bound structure introduces lipid-like density in both bilayer leaflets. While a minor offset of TMD2 was seen, (Figure 1B) the movement is not as pronounced as that seen for the lipid-embedded structures (Figures 1B and 2E). Thus, the two ABCA7 studies agree on the conserved TMD–NBD–RD engine and detergent state architecture but differ in what they reveal about membrane integration and cavity geometry.

Together, these observations suggest that membrane-integrated behavior with a through-passing bilayer patch is currently directly established only for nanodisc-embedded ABCA7, where the cleft appears to accommodate headgroup movement and leaflet distortion, creating a potential substrate corridor across or within the bilayer. This path vanishes upon NBD closure, fitting a model in which membrane deformation is transient and ATP-gated. What remains is an “exit pocket” likely able to accommodate lipid molecules primed for extrusion to the ECDs. This further suggests that in addition to lipid translocation through flipping and/or extrusion, ABCA transporters could potentially gate access to internalized lipid-lined conduits. It remains unclear whether headgroups cross the midplane or reorient within leaflets, but the structural basis for bilayer perturbation is clear. In the model proposed, ABCA7 could act both as a cross-leaflet lipid translocator and lipid extruder from external leaflet to the ECD. The reliance on detergent micelles may have enforced an artificial bilayer truncation, eliminating the continuous lipid sheet that shapes the TMD vestibules and conformational landscape in lipid-embedded preparations.

## The role of accessory domains and potential apolipoprotein interactions

If lipids emerge from the TMD vertically, the ECDs are natural endpoints. ABCA transporter structures resolved regardless of detergent or nanodisc formulations have revealed extra-proteinaceous density in the ECD tunnel attributed to lipid/sterol entities, although this remains speculative owing to their generally diffuse nature and lack of resolution. In the case of ABCA1, density features within ECD tunnels were shown to be absent in structures of cholesterol-binding deficient mutants—supporting transient lipid sequestration [93]. However, whether this represents true cholesterol capture or nonspecific hydrophobic aggregation remains unresolved and its physiological relevance remains ambiguous. ABCA3 ECDs are smaller, while ABCA7 mimics ABCA1. These tunnels are ∼60 Å in height and lined with hydrophobic and glycosylated residues. Whether they transiently bind lipids en route to acceptors like apoA-I lacks direct mechanistic validation. The convergence of structural and biochemical data has been interpreted as consistent with an ECD-mediated lipid hand-off model, in which the apolipoprotein interface completes the extrusion cycle, but how this interaction couples to bilayer deformation remains an open question.

The ECDs for ABCA7 and ABCA1 are known to interact with apolipoproteins, a fundamental property of wide-ranging physiological relevance [10,13,107,108]. Despite decades of biochemical work, the mechanistic basis for this purported interaction remains unknown, with at least nine possible models invoked, spanning direct trafficking to endosomal recycling, as comprehensively summarized elsewhere [109]. Each posits transient lipidic intermediates, but none capture the bilayer context that governs them. Thus, the multiplicity of models is itself evidence that detergent or simplified membranes cannot reproduce the coupled lipid–protein transitions ABCA transporters may require for their physiological function.

Compared with the ECDs, a lot less is known about the cytosolic RDs. Their structural role is evident in imparting domain swapping at the intracellular side that, in combination with the domain-swapped ECDs, can more tightly couple the two halves of the transporters. Considering the dominance of rigid-body motions in large-scale domain movement, changes in RD positioning could plausibly be transferred to the ECDs. In fact, mutations in the ABCA1 RD have been proposed to lead to changes in the ECD–apolipoprotein interaction, though the exact nature of this remains speculative. Other plausible roles could be stabilizing and regulating nucleotide binding by the NBDs and serving as sites for phosphorylation, with evidence linking increased ABCA1 stability to kinase treatment. Overall, these main ABCA transporter accessory elements, the large ECDs and short cytosolic RDs, provide the structural interfaces for potential apolipoprotein engagement and inter-domain communication.

## Conclusions

**Figure 3:**
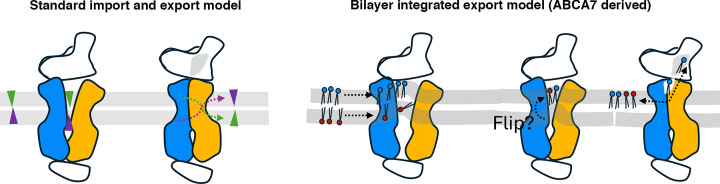
Proposed substrate transport mechanisms for ABCA transporters (**A**) The standard model invoked for both import and export substrates (green and purple triangles, respectively). Interleaflet substrate “flipping” is denoted by dashed arrows colored similarly and inverted triangle orientation. Each black-outlined component of the transporter represents a single RBG for simplicity. Gray bars represent bilayer leaflets. (**B**) The membrane-integrated transport model based on structures of lipid-embedded ABCA7 introduces membrane lipid composition and structure as key components of the transport mechanism. Lipids are depicted as double black lines with red and blue filled circles representing different head groups. Dashed arrows indicate lipid movement directionality.

The ABCA transporter work highlighted here defines conserved, nucleotide hydrolysis-coupled TMD–NBD core rearrangements that, in the case of ABCA7, reshape and engage the surrounding bilayer. Whether this applies to the rest of the family, including ABCA1, with which ABCA7 shares significant sequence and structural homology, will require a systematic analysis of the full panel of ABCA transporters in identical lipid-embedded states in the presence of different substrates and nucleotides. In the absence of a unified mechanistic framework, Figure 3 presents the two primary models emerging from the structural biology data for ABCA transporters to date. In the simplified transport model used extensively in the ABCA literature to describe both import and export reactions (Figure 3A), substrates from either leaflet access a single “open” conformation and are displaced to the opposite leaflet after NBD closure. The model therefore treats leaflet identity and directionality as interchangeable, and the underlying geometric constraints of the bilayer are not part of the mechanism. By contrast, the membrane-integrated model is an explicit export mechanism derived from lipid-embedded ABCA7 structures (Figure 3B). It incorporates local bilayer deformation, transient passage of an ordered leaflet segment through the TMD cavity, and extrusion toward the ECD through a structurally identifiable exit path as well as potential flipping of inner leaflet lipids to the outerleaflet. Here, membrane-integrated transport is not a static lock-and-key exchange but a dynamic negotiation between a shape-shifting key and a pliant lipidic lock, each adjusting within the fluctuating bilayer. Whereas the standard model accommodates import or export only by diagrammatic inversion, the ABCA7-derived membrane-integrated model provides a physical basis for lipid selectivity, leaflet bias, and coupling of domain motion to bilayer mechanics.

Family-level distinctions within different ABCA transporters arising from the ECDs and their coupling to the membrane and interaction partners reframe ABCA transporter evolution as an ECD-driven adaptation of the above-mentioned conserved bilayer-coupled engine, where ECD1 modifications dictate how the protein–bilayer interface accommodates apolipoproteins, matrix contacts, or leaflet reshaping. Recognizing these ECD1-based lineages clarifies why transporters with highly similar transmembrane machineries show distinct physiological behaviors—and why a single “flipping” model may not fully describe them all. Local membrane composition, including headgroup asymmetry and lipid packing, varies widely across tissues as extensively reviewed here [110–113], among others, and likely contributes to functional divergence across ABCA paralogs. ABCA family members can therefore be viewed as membrane-integrated machines in which lipid, protein, and nucleotide chemistry act as a single mechanical system. In this sense, the bilayer-integrated model provides a useful working framework that reconciles structural, biochemical, and cellular data for ABCA7 and offers testable hypotheses for other family members as lipid-embedded structures become available.

While pioneering structural work on ABCA1 provided the indispensable architectural framework for this family, enabling subsequent refinement and reinterpretation across paralogs, several key mechanistic uncertainties persist. For example, it is unclear whether substrate translocation is bidirectional across ABCA4, A1, and A7; how lipid specificity is achieved; whether headgroups truly cross the midplane or slide within a leaflet; and what functional role the cytosolic RDs play—stabilizing intermediates, gating activity, or linking transporter halves. At the extracellular side, it remains unknown whether the ECDs sequester or chaperone lipids and how these transient lipid-bound states couple to apolipoprotein interaction. A mechanistic understanding of these key features is essential for addressing the exact roles transporters like ABCA7, mechanistically linked to Alzheimer’s disease, play in disease progression and holds tremendous potential for opening new avenues for the development of novel diagnostic and therapeutic tools. Finally, several of the ABCA transporters remain poorly studied, among them the most intriguing perhaps being ABCA13—the largest and least understood member—which remains a mechanistic and physiological outlier despite clear evidence of expression and structural organization.

## Perspectives

- ABCA transporters are central to multiple fundamental physiological pathways spanning cholesterol efflux and HDL biogenesis, pulmonary surfactant production, photoreceptor retinoid clearance, lipid homeostasis in microglia, and barrier formation in skin. Dysfunctional variants in ABCA1, ABCA3, ABCA4, ABCA7, and ABCA12 contribute directly to major human pathologies, including atherosclerosis, neonatal respiratory distress, Stargardt disease, ichthyoses, and Alzheimer’s disease—while ABCA13 and other lesser-characterized paralogs are increasingly implicated in psychiatric and neurodevelopmental conditions.
- Recent structures of ABCA1/3/4/7 have yielded a common architectural framework and conformational spectrum likely shared by other members of the family. However, the predominant mechanistic interpretations do not fully account for the bilayer's role in shaping transporter energetics. Emerging evidence from lipid-embedded preparations of ABCA7 indicates that membrane integration can alter cavity geometry, leaflet access, and extrusion pathways, suggesting that substrate transport is tightly coupled to local lipid environment, but several mechanistic questions about substrate specificity and directionality, apolipoprotein interactions, and bilayer coupling, among others, remain open. At present, this membrane-integrated behavior is structurally established only for ABCA7, and its generality across other family members will require comparable lipid-embedded datasets.
- Addressing these mechanistic questions remains difficult because cryo-EM, for all its power, offers limited chemical specificity: lipid-like densities seldom rise above noise without artificial stabilization or halogenation, which itself perturbs bilayer mechanics. Even when visible, such features may represent static echoes of transient membrane rearrangements rather than bona fide bound substrates. Progress will require tools capable of resolving inter-domain coupling and lipid engagement within native bilayers at higher fidelity, with an integration of conformation-specific nanobodies, native mass spectrometry, and cryo-electron tomography holding promise for capturing intermediate states in these complex, membrane-integrated machines.

## Open Access

Open access for this article was enabled by the participation of the University of Minnesota in an all-inclusive *Read & Publish* agreement with Portland Press and the Biochemical Society under an agreement with the Big Ten Alliance.

## Abbreviations

APP: amyloid precursor protein
Aβ: amyloid-beta
apoA1: apolipoprotein A1
ABC: ATP-binding cassette
ECD: extracellular domain
GWAS: genome-wide association study
NBDs: nucleotide-binding domains
RDs: regulatory domains
RBGs: rigid body groups
TMDs: transmembrane domains
